# Individual Cognitive Stimulation Therapy for dementia (iCST): study protocol for a randomized controlled trial

**DOI:** 10.1186/1745-6215-13-172

**Published:** 2012-09-22

**Authors:** Martin Orrell, Lauren A Yates, Alistair Burns, Ian Russell, Robert T Woods, Zoe Hoare, Esme Moniz-Cook, Catherine Henderson, Martin Knapp, Aimee Spector, Vasiliki Orgeta

**Affiliations:** 1 Mental Health Sciences Unit, University College, London, UK; 2 Department of Old Age Psychiatry, University of Manchester, Manchester, UK; 3 School of Medicine, Swansea University, Swansea, UK; 4 Dementia Services Development Centre Wales, Bangor University, Bangor, UK; 5 North Wales Organisation for Randomised Trials in Health (& Social Care), University of Bangor, Bangor, UK; 6 Institute of Rehabilitation, University of Hull, Hull, UK; 7 London School of Economics and Political Science, London, UK; 8 Institute of Psychiatry at King’s College London, London, UK; 9 Department of Clinical Psychology, University College London, London, UK

## Abstract

**Background:**

Improving the quality of care for people with dementia and their carers has become a national priority in many countries. Cognitive Stimulation Therapy (CST) groups can be beneficial in improving cognition and quality of life for people with dementia. The aim of the current study is to develop and evaluate a home-based individual Cognitive Stimulation Therapy (iCST) programme for people with dementia which can be delivered by their family carer.

**Methods:**

This multi-centre, pragmatic randomised controlled trial (RCT) will compare the effectiveness and cost-effectiveness of iCST for people with dementia with a treatment as usual control group. The intervention consists of iCST sessions delivered by a carer for 30 minutes, 3 times a week over 25 weeks.

For people with dementia the primary outcome measures are cognition assessed by the ADAS-Cog, and quality of life assessed by QoL-AD. For carers, quality of life using the SF-12 is the primary outcome measure. Using a 5% significance level, comparison of 306 participants will yield 80% power to detect an effect size of 0.35 for cognition as measured by the ADAS-Cog, and quality of life as measured by the QoL-AD. Quality of life for the carer will be measured using the SF-12. The trial will include a cost-effectiveness analysis from a public sector perspective.

**Discussion:**

The UK Department of Health has recently stressed that improving access to psychological therapies is a national priority, but many people with dementia are unable to access psychological interventions. The development of a home-based individual version of CST will provide an easy to use, widely available therapy package that will be evaluated for effectiveness and cost-effectiveness in a multi centre RCT.

## Background

Caring for people with dementia has an enormous impact on health and social care services and on family carers [1]. The cost of dementia in the UK is over £17 billion a year [2]. With the number of people living with dementia expected to double in the next thirty years, improving the quality of care for people with dementia and their carers has become a national priority [1]. In the UK there is growing recognition that psychological therapies for dementia should be more widely available. Indeed the National Service Framework for Older People emphasises the use of non-pharmacological management strategies, such as mental stimulation for dementia, and the UK Department of Health has identified improving access to psychological therapies as a priority [3].

Cognitive Stimulation Therapy (CST) is an evidence-based approach for people with dementia developed following Cochrane reviews of several psychosocial therapies for dementia, primarily reality orientation (RO) [4]. RO involves the presentation and repetition of orientation information, such as the date, day and weather [5]. This may take place intensively throughout the day, or in regular structured group meetings. Benefits of RO noted in the Cochrane review [4] included improved behaviour and cognition. In addition the need for a more detailed and ongoing programme of orientation activities and large scale multi-centre trials to evaluate this approach was identified. Spector *et al*. found that participating in CST improved quality of life and cognition for people with dementia [5]. CST may also be more cost-effective than anti-dementia drug treatments [6]. CST is currently the only non-pharmacological therapy recommended by the National Institute for Health and Clinical Excellence (NICE) guidelines [7] to improve cognition in people with mild to moderate dementia. A pilot study of an extended programme of maintenance CST found a significant improvement in cognitive function for those receiving maintenance CST, suggesting that benefits could be maintained by weekly sessions for at least 6 months [8]. Olazaran*et al*. also found that CST groups had long-term cognitive benefits for people with dementia [9]. The Maintenance CST programme (comprising 14 CST sessions over 7 weeks plus an additional 24 weekly maintenance CST sessions) and accompanying manual have now been further developed as part of the SHIELD study [10] and evaluated in a randomised controlled trial (RCT). The results of the Maintenance CST trial are expected soon.

The use of group CST is growing rapidly in the UK and internationally, yet many people with dementia may be unable or unwilling to participate in group CST. This could be because they do not want to go out, or because they have restricted mobility or health issues that prevent them from getting out; they may choose not to participate in group-based activities, or groups may not be running in their local area. To assess the acceptability of an individualised CST programme we surveyed care staff attending CST training sessions and carers from the charity *For Dementia*, and we spoke to carers and people with dementia. There was a consensus from people with dementia and family carers that individualised CST should be a high priority because it was likely to be very useful. Comments included 'sounds terrific', 'could bring the carer and person with dementia closer together', 'good for people who won't go out', and 'definitely needed as a useful alternative to medication'. Taken together the evidence suggests that a large-scale trial of iCST for dementia in the UK is feasible, likely to be effective and should be a high priority for research.

Few studies have focused on the use of cognitive stimulation programmes in the home environment. In a pilot study, Moniz-Cook *et al*. [11] found that a home-based memory management programme involving the family carer led to improvements in memory in the person with dementia, improvements in carer wellbeing, and a reduction in care home admissions at 18 months follow-up. Similar benefits in cognition in people with dementia and carer wellbeing have been reported in studies by Quayhagen*et al*. [12] and Quayhagen and Quayhagen [13]. Onder *et al*. [14] carried out a study of patients with Alzheimer’s disease (AD) taking cholinesterase inhibitors. The intervention consisted of a standardised programme of RO delivered by the family carer in the home for 30 minutes, three times a week over 25 weeks. Alongside training, carers were given a manual, specific schedules for each session, and guidance on how to deliver the sessions. The experimental group receiving the intervention improved relative to the control group on both the Mini Mental State Examination (MMSE) and the Alzheimer’s Disease Assessment Scale – Cognitive subscale (ADAS-Cog).

The primary aim of the proposed trial is to investigate whether individual home-based CST benefits cognition and quality of life in people with dementia and improves carer well-being. Based on previous research findings, we hypothesize that people with dementia receiving iCST will show improvements in cognition and quality of life. A secondary aim of the trial is to explore the costs of those receiving iCST compared to a control group, and to investigate whether iCST is cost-effective.

## Methods

### Design

**Figure 1:**
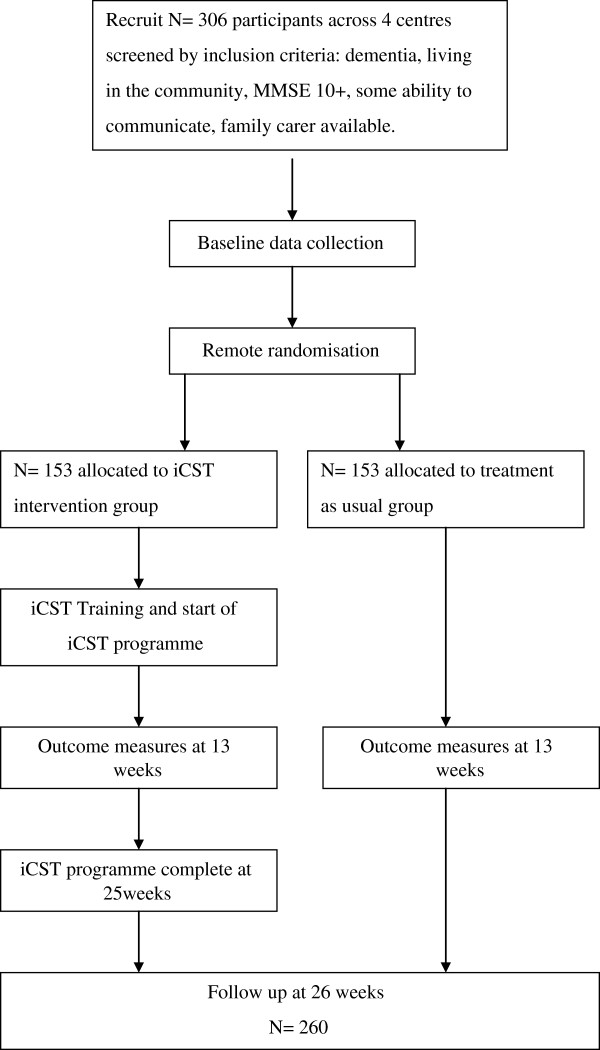
Flow of participants through the iCST randomised controlled trial.

The design is a multi-centre, single blind, randomized, two-treatment arm (iCST over 25 weeks vs. treatment as usual, or TAU), controlled clinical trial (Figure 1). After recruitment and baseline assessments, pairs of people with dementia and their carer are randomly allocated into either the treatment group (receiving three 30-minute weekly sessions of iCST delivered by the carer for 25 weeks) or control group (receiving treatment as usual for 25 weeks). Primary and secondary measures are completed at baseline (T0) before the iCST programme, first follow-up at 13 weeks after baseline (T1) and second follow-up and primary endpoint at 26 weeks after baseline (T2).

### Sample size

Cognition (ADAS-Cog) will be the primary outcome measure. The group CST study by Spector *et al*. [5] had an effect size (standardised mean difference, or SMD) of 0.32.The Spector *et al*. Cochrane Review of RO [4] found an SMD of 0.58. The Maintenance group CST study [8] found an SMD of 0.68 compared to TAU. A recent Cochrane Review of cognitive stimulation found an SMD of 0.37 [15]. Taking a conservative estimate, SMD relative to TAU for iCST is estimated to be at least 0.35. In order to detect an SMD for iCST of 0.35 on the ADAS-Cog with 80% power at a 0.05 (two-sided) significance level, and assuming 15% attrition, a sample size of 306 people with dementia will be required. Experience in previous trials including the CST trial, the needs in care homes trial [16], and the activities in care homes trial [17], indicates a 12 to 15% loss to follow-up (7 to 10% excluding deaths) is likely. To safeguard loss to follow-up, standard procedures to maximize the follow-up sample will be applied. These will include regular contact with carers via telephone, letters (for example, reminders for assessment or training appointments) and email, if requested by the carer.

### Participants

Recruitment to this trial will take place in a variety of community settings including community mental health teams for older people (Chats), memory clinics, outpatient clinics, day centres and voluntary sector organisations such as Age Concern and the Alzheimer’s Society. Some participants in both the intervention and TAU groups will be taking anticholinesterase inhibitors; in these cases participants will continue taking them throughout the study. Participants will be screened for eligibility using the Spector *et al*. [4] standardised criteria for psychological treatment of people with dementia. Participants must meet the Diagnostic and Statistical Manual of Mental Disorders (DSM)-IV criteria for dementia, have dementia of mild to moderate severity (MMSE ≥10), have some ability to communicate and understand, and be able to see and hear well enough to participate in activities. In addition, they must have a carer who will be available to deliver the intervention, live in the community, and have no major illness which could affect participation. Participants may only enter the study after giving informed consent in accordance with the provisions of the Mental Capacity Act 2005 [18].

### Randomisation

Randomisation will occur after screening and baseline assessments. The allocation ratio for randomisation is 1:1, into either the intervention group or control group (TAU). Participants will be stratified by centre (London, Bangor, Hull or Manchester) and whether they are taking anticholinesterase inhibitors, to ensure even distribution of the sample between the treatment and control groups. Registered participants will be randomised by the web-based randomisation service managed by North Wales Organisation for Randomised Trials in Health (NWORTH), an accredited UK Clinical Trials Unit. The randomisation algorithm is a dynamic adaptive method that ensures balance overall, within each stratification variable and within each stratum. This allows sequential randomisation of participants, minimising selection bias while maintaining an acceptable level of balance [19]. Although participants cannot be blinded to their treatment allocation, researchers carrying out follow-up assessments will be blinded to the treatment condition. Our experience shared by similar projects is that participants may occasionally inadvertently reveal their allocation to researchers. In order to reduce this effect, participants will be given explicit reminders before the experimental visit and self-reported measures will be used wherever feasible. Assessors will record their impression of which arm of the trial each participant belongs to, and their confidence in that prediction. This will enable us to conduct a retrospective estimation of the integrity of blinding, to test whether inadvertent loss of blinding leads to bias, and to adjust for any bias detected.

### Intervention

The iCST programme is based on a modified CST manual, the recent Cochrane review of cognitive stimulation [15], Onder’s programme [14] and consultation with carers and people with dementia. iCST will be delivered by a carer in regular contact with the person with dementia for 30 minutes, three times a week over 25 weeks. The iCST programme comprises 75 iCST sessions consisting of structured cognitive stimulation through themed activities (for example, number games, associated words) (Table 1) tailored to the ability, interests and needs of the individual. Carers will receive the iCST instructional Manual and Activity Workbook for use during the programme. The Manual provides guidance on how to run the sessions, the key principles of iCST and ideas for activities for each session. The Activity Workbook contains paper-based resources for activities suggested in the manual. Carers will also be provided with the iCST kit, which will include additional resources such as a deck of cards, set of dominoes, magnifying card, sound activity compact discs (CDs), set of boules, and world and UK maps. A first draft of the iCST Manual, Activity Workbook and iCST kit will be developed by the research team, and presented to people with dementia and carers in interviews and focus groups (adhering to Medical Research Council (MRC) guidance [20]). The purpose of consultation with service users is to ensure that the Manual and Activity Workbook are easy to use, describe meaningful activities, are appropriately tailored to people with mild and moderate dementia, and that the iCST kit contains suitable items. The iCST package will be further evaluated using the Delphi process of consensus methodology, in line with guidelines for consensus methods in medical and health services research [21]. A feasibility study with a sample of 20 people with dementia and their carers will be carried out prior to the main RCT. A final draft of the iCST package incorporating findings from the feasibility study will be produced for use in the full trial.

**Table 1:** **Individual cognitive stimulation therapy** (**iCST) themes**

**iCST Session theme**	**Session number**
My life	1, 2, 45, 46
Current affairs	3, 4, 57, 58
Food	5, 6, 55, 56
Being creative	7, 8, 63, 64
Number games	9, 10, 71, 72
Quiz games	11, 12, 75
Sounds	13, 14, 51, 52
Physical games	15, 16, 49, 50
Categorizing objects	17, 18, 65, 66
Household treasures	19, 20
Useful tips	21, 22, 47, 48
Thinking cards	23, 24
Visual clips discussion	25, 26
Art discussion	27, 28, 43, 44
Faces/scenes	29, 30, 59, 60
Word games	31, 32, 41, 42, 73, 74
Slogans	33, 34
Associated words/discussion	35, 36, 61, 62
Orientation	37, 38, 67, 68
Using money	39, 40, 69, 70
Childhood	53, 54

### Treatment adherence, carer training, and support

Previous research suggests that in order to investigate treatment process variables, and to ensure that psychosocial interventions can be replicated, it is necessary to have precise descriptions of treatment components, and to ensure that the treatment delivered was indeed the treatment intended. We will follow previous studies [22] applying the treatment integrity model, developed and expanded by Lichstein, Riedel and Grieve [23].Carers will receive standardised training either in their homes or in a group setting, according to which is most convenient for the carer. The training that researchers will provide to carers will focus on how to use the iCST Manual and Activity Workbook, implementing the key principles of CST and problem solving strategies. In the training session the researcher will show clips of good practice in CST from the *Making a Difference 2* training digital video disc (DVD). The DVD was developed as part of the Maintenance CST trial [10]. If the training session is home-based, the carer will be invited to deliver the first session with support from the researcher, who will provide assistance and feedback. Carers will receive the iCST Manual, Activity Workbook and kit as part of a training and set-up visit. Researchers will be guided by a standardised treatment protocol detailing training procedures and support provided. During the trial carers will receive up to ten hours of support over six months, including telephone support (initially weekly) and two visits from the unblinded researcher. In the event that the family carer is unable to continue delivering iCST, another appropriate carer can be substituted.

### Usual care

The control group will receive TAU, which may vary between and within centres and change over time, therefore the study will evaluate the additional effects of iCST. In terms of treatment we would expect most people with mild to moderate AD will either be on, or have been considered for, cholinesterase inhibitor medication. The Client Service Receipt Inventory (CSRI) will enable us to accurately record use of drugs and services across the two groups and any changes that occur. In general, the services offered to this group will also be available to those in the active treatment group, so we will be examining the additional effects of iCST.

### Resource use

The CSRI [24] will allow us to record the utilisation of services and the interventions received during the study, and the support provided by carers, as well as the use of cholinesterase inhibitors and other psychiatric medications such as antipsychotics and antidepressants. Data will also be collected on the inputs required to deliver the intervention.

### Ethical approval

Ethical approval was obtained through the Multi-centre Research Ethics Committee (ref no.10/H0701/71), and the study is registered as a clinical trial (ISRCTN 65945963). There appear to be no documented harmful side effects from participating in CST groups, and no serious adverse reactions were apparent in the CST study [5]. Prospective participants will be fully informed of the potential risks and benefits of the project. A reporting procedure will be in place to ensure that any serious adverse events are reported to the Chief Investigator. Participants will be in the mild to moderate stages of dementia, and would therefore generally be expected to be competent to give informed consent for participation, provided that appropriate care is taken to explain the research. Where the participant’s level of impairment increases, so that he/she is no longer able to provide informed consent, the provisions of the Mental Capacity Act [18] will be followed, with the family caregiver as a consultee.

### Outcome measures

#### Primary outcome measures for the person with dementia

Cognition will be measured using the ADAS-Cog [25], which consists of 11 tasks assessing disturbances of memory, language, praxis, attention and other cognitive abilities, referred to as the core symptoms of AD, with good reliability and validity [26].Quality of life will be measured using the Quality of Life Alzheimer’s disease Scale (QoL-AD) [27] which consists of 13 domains of quality of life. The measure is recommended by the European consensus on outcome measures for psychosocial interventions in dementia [28].

#### Secondary outcome measures for the person with dementia

Quality of life will also be measured with the Dementia Quality of Life (DEMQOL) scale [29]. The scale uses self-rated reports of quality of life across five domains administered to the person with dementia by a trained interviewer. It has high internal consistency, acceptable inter-rater reliability and good concurrent validity, with moderate associations with the QoL-AD [30]. It is included as a quality of life scale and a utility measure since an algorithm is now available to convert the DEMQOL and DEMQOL-proxy into utility scores [31].Behaviour will be assessed using the Neuropsychiatric Inventory (NPI) [32]. The NPI measures 10 behavioural disturbances occurring in dementia patients. It is reported to be both valid and reliable [33]. Functional ability of the person with dementia will be assessed using the Bristol Activities of Daily Living Scale (BADLS) [34], which is a carer-rated instrument assessing items rated as important by carers in 20 daily-living abilities. The measure shows sensitivity to change in people with AD taking anticholinesterase medication, and is associated with changes in the ADAS-Cog [35].Depressive symptoms will be measured by the Geriatric Depression Scale (GDS-15) [36], comprising 15 easy-to-use items. The GDS-15, although principally a self-rating scale, may be used as an observer-administered scale, with acceptable sensitivity and specificity in people with mild to moderate dementia [37].Quality of the carer-patient relationship (QCPR) [38] will be assessed by both the carer and the person with dementia. The QCPR is a measure of relationship quality, comprising 14 items designed to assess warmth, levels of conflict and criticism in the caregiving relationship. Previous studies have shown that the QCPR has good internal consistency and concurrent validity [38].

#### Primary outcome measures for the carer

Health-related quality of life will be measured using the Short Form-12 Health Survey (SF-12) [39]. The SF-12 is a comprehensive, psychometrically sound, and efficient measure of health which includes eight concepts commonly represented in health surveys: physical functioning, role functioning, physical pain, general health, vitality, social functioning, emotional and mental health.

#### Secondary outcome measures for the carer

Anxiety and depression will be assessed using the Hospital Anxiety and Depression Scale (HADS) [40], a widely used measure of self-reporting consisting of 14 questions, validated in several age groups, which identifies caseness for clinically significant depression and anxiety [41].Self-reported health related quality of life will be measured using the EQ-5D [42]. The EQ-5D is a self completed measure yielding a simple descriptive profile and a single index value for health status. It has been used in a wide range of study populations (Pickard *et al*., 2007 [43]; Dyer *et al*., 2010 [44]). Resilience in carers will be measured with the Resilience Scale (RS-14) developed by Wagnild and Young [45]. In the shorter version of this scale participants are asked to respond to each item by either agreeing or disagreeing with each statement, with higher scores indicating stronger resilience. Previous studies have shown that the measure demonstrates high internal consistency and construct validity [46].

#### Economic measures

Care and support levels will be assessed using the CSRI [24], adapted for this study and used extensively in studies of mental health and dementia. The CSRI gathers comprehensive data on accommodation, medication and services received, as well as details of unpaid support from carers, and wider carer economic impacts. A form for monitoring treatment adherence will be devised for this study and will include questions on the amount of time required from professionals and carers to support the delivery of the training package. Costs of care and support can be estimated from these service-use data by applying relevant, nationally generalisable unit costs, drawing on the National Health Service (NHS) reference costs [47] and the annual Personal Social Services Research Unit (PSSRU) volume [48]. The costs of delivering the training package (excluding costs of the initial development and testing of the package) will be calculated from the perspective of commissioners (NHS, local government) and also in terms of costs to carers of their time. Costs will be reported in aggregated and disaggregated form (NHS overall, local government, society as a whole) to show total programme cost, cost per participant (person with dementia), and cost per participant-carer pair. Cost effectiveness will be computed in a number of different ways as the difference in costs between the iCST and control group over the trial period, divided by the difference in outcomes (cognition, quality of life, or QALYS). See the Economic evaluation section below for details.

### Analysis

An intention-to-treat analysis will be carried out, in that all available data will be included. A method of multiple imputation using a linear regression model will be used where needed for imputing missing data. The sample size calculations are based on the numbers estimated to be available at the study endpoint, 6 months after randomisation. Analysis of covariance will be used to adjust for baseline differences that may influence outcome variables. Variables to be considered in the model will include, among others, gender and age. Analyses will consider the evaluation 6 months after randomisation as the primary endpoint in evaluating the effectiveness of iCST. Further model definition will be provided in the statistical analysis plan.

### Economic evaluation

The main economic evaluation will be a cost-effectiveness analysis (CEA),first from a health and social care perspective, and second, from a societal perspective. Service-use data, and information on unpaid carer support will be collected using an adapted CSRI, and then converted into estimates of costs by applying nationally generalisable unit cost data.

Carer inputs will be costed in two ways, using either a replacement cost assumption or an opportunity cost assumption (Koopmanschap, 2008 [49]; Pritchard, 2000 [50]). The primary CEA will measure effectiveness using the ADAS-Cog; further analyses will look at other outcomes, particularly quality of life as measured by the QoL-AD and QALYs generated from the DEMQOL and DEMQOL-proxy by applying societal weights [31]. The use of QALYs will allow bodies such as NICE to make recommendations about the use of health and social care resources so as to achieve the greatest impact from given budgets; cost-per-QALY calculations are increasingly used in health systems in pursuit of greater allocative efficiency (Smith and Richardson, 2005 [51]; Rawlins and Culyer, 2005 [52]). Each such CEA will be conducted from a health and social care perspective, and then from a societal perspective.

Cost-effectiveness acceptability curves will be plotted, generated from the net benefit approach and using bootstrap regression for a range of values of willingness to pay for incremental primary outcome measure changes and QALY gains. CEACs are widely employed as a way to quantify and graphically represent uncertainty in economic evaluation studies of health care technologies [53]. The economic evaluation will be fully integrated into the main outcome evaluations. Sensitivity analyses will be carried out to determine whether changes in the values of the main parameter estimates affect the results of the analyses.

## Discussion

This is an innovative RCT that evaluates the effectiveness and cost-effectiveness of individual CST for people with dementia and their carers. The development of carer-led therapies could ease pressure on local services, which are in great demand but often severely limited. In 2009, the UK National Audit Office reported that CST was available in 29% of CMHTs for older people [54] and a home-based version of CST, could help people with dementia having limited access to CMHT services. By placing emphasis on working with the person with dementia and family carer together, the study meets the current demand for relationship-centered care and will provide the opportunity to explore the dynamics of carer-led therapies compared to professional-led therapies. We anticipate that actively involving carers in the delivery of a therapy package will be empowering, and will also have a positive impact on their well-being.

The NICE-Social Care Institute for Excellence (SCIE) guidelines [7] on the management of dementia offer few evidence-based recommendations on psychosocial approaches, due to a paucity of high quality RCTs. The current RCT is the first study to assess the cost-effectiveness of an individualised carer-led cognitive intervention in dementia.

The potential benefits of iCST include improved well-being for people with dementia and their carers, and economic and social benefits such as reduced costs of care and delayed institutionalisation. iCST can also be offered in combination with anti-dementia medication, and also provides an option for those unsuitable for, or unwilling to take medication. However, the success of iCST will be heavily dependent on family carers being motivated and able to invest time to adhere to the programme. The number of commitments (for example, hospital appointments) and responsibilities (for example, household upkeep) carers have on an everyday basis, and how well they are coping with caring for their relative with dementia may have an impact on adherence to the programme. In addition, carer’s confidence in their ability to adopt a therapeutic role delivering iCST sessions may also affect the success of the programme, as some may consider this to be a role best occupied by healthcare professionals and day centre staff. Providing a high quality interactive training package and adequate support for carers will be key to avoiding or minimising the impact of these potential issues.

A longer term follow up would be beneficial to examine rates of institutionalisation and cost of care in the months or years following completion of the iCST programme, to determine whether iCST plays a role in delaying institutionalisation and reducing the cost of care beyond the duration of taking part in the sessions. The trial results will contribute to future practice guidelines and, if successful, the iCST programme could be widely used across the UK and internationally, and become the gold standard for individual cognitive stimulation-based interventions in dementia.

### Trial status

The trial is ongoing.

## Abbreviations

AD: Alzheimer’s disease; ADAS-Cog: Alzheimer’s Disease Assessment Scale - Cognitive Subscale; CMHT: community mental health team; BADLS: Bristol Activities of Daily Living Scale; CEA: cost effectiveness analysis; CEAC: cost effectiveness acceptability curve; CSRI: Client Service Receipt Inventory; CST: cognitive stimulation therapy; DEMQoL: Dementia Quality of Life; DEMQoL-proxy: Dementia Quality of Life Proxy; DSM-IV: Diagnostic and Statistical Manual of Mental Disorders; DVD: digital versatile disc; EQ-5D: European Quality of Life - 5 Dimensions; GDS: Geriatric Depression Scale; HADS: Hospital Anxiety and Depression Scale; iCST: individual cognitive stimulation therapy; maintenance CST: maintenance cognitive stimulation therapy; MMSE: Mini Mental State Examination; NHS: National Health Service; NPI: Neuropsychiatric Inventory; NICE: National Institute for Health and Clinical Excellence; NICE-SCIE: National Institute for Health and Clinical Excellence- Social Care Institute for Excellence; NWORTH: North Wales Organisation for Randomised Trials in Health; PSSRU: Personal Social Services Research Unit; QALY: quality adjusted life year; QCPR: quality of the carer patient relationship; QoL-AD: Quality of Life Alzheimer’s Disease; RCT: randomised controlled trial; RO: reality orientation; RS-14: Resilience Scale; SF-12: Short Form-12 Health Survey; SHIELD: Support at Home, Interventions to Enhance Life in Dementia; SMD: standardised mean difference; TAU: treatment as usual; T0: baseline; T1: first follow-up; T2: final follow-up.

## Competing interests

The authors declare that they have no competing interests.

## Authors’ contributions

MO, RTW and AS developed the original concept of the trial, and MO drafted the original protocol. IR developed the design and methodology; ZH developed the analysis plan; MK and CH developed the health economic component. LY and VO adapted the trial proposal as a protocol paper; LY, AS, MO and VO have contributed to the development of the iCST approach; all authors reviewed and commented on drafts of the protocol and paper. All authors read and approved the final manuscript.
